# Atomic Detail from Disordered Regions: QM/MM-Based Real-Space Reconstruction ofLoops, Rotamers, and Protonation States in X-ray/Cryo-EM Density

**DOI:** 10.1063/4.0001026

**Published:** 2025-10-27

**Authors:** Lance M Westerhoff, Oleg Borbulevych

**Affiliations:** QuantumBio Inc, State College, PA, USA

## Abstract

Protein flexibility is central to function—governing allosteric regulation, catalysis, signal transduction, and drug binding. Yet, this flexibility is often underrepresented in structural models derived from X-ray or Cryo-EM experiments, where flexible loops, alternate protonation states, and mobile side chains are frequently unresolved or omitted. These omissions limit the accuracy of molecular dynamics (MD), free energy calculations, and mechanistic insights. In this talk, we present a density-driven, quantum-informed protocol for reconstructing disordered and flexible regions of macromolecular structures, enabling more complete and chemically accurate models suitable for dynamic simulation and predictive design.

Implemented in the DivCon Discovery Suite, our approach rebuilds missing backbone loops using φ/ψ sampling with geometry and density-aware filtering. Real-space refinement is applied at each stage—backbone closure, rotamer placement, and protonation state selection—using QM, MM, or hybrid QM/MM Hamiltonians combined with X-ray or Cryo-EM electron density gradients. Z-score of the difference density (ZDD) is used throughout to evaluate and select the most experimentally consistent conformations. The workflow also includes tautomer/protomer determination and hydrogen-bond network-aware protonation state sampling.

We demonstrate how this method restores conformational diversity essential for understanding loop gating, ligand-induced rearrangements, and proton-coupled mechanisms. Case studies reveal improved structural stability during simulations, stronger correlation between ligand and loop motion in MD, and enhanced accuracy in binding free energy predictions. These refinements not only support more faithful molecular “movies” of protein function but also improve the quality of AI/ML training datasets used for structure and property prediction.

This work highlights the importance of recovering conformationally dynamic features from density data to enable flexible, functionally relevant, and therapeutically actionable protein models.

